# The vulnerability of RB loss in breast cancer: Targeting a void in cell cycle control

**DOI:** 10.18632/oncotarget.25797

**Published:** 2018-07-24

**Authors:** Erik S. Knudsen, Eldad Zacksenhaus

**Affiliations:** Erik S. Knudsen: Department of Molecular and Cellular Biology, Roswell Park Cancer Center, Buffalo, New York, USA; Eldad Zacksenhaus: Toronto General Research Institute, University Health Network, Toronto, Ontario, Canada

**Keywords:** triple negative breast cancer, targeted therapy, RB1, CHK1, CDC25

**Introduction:** The retinoblastoma tumor suppressor (RB) is an important regulator of the cell cycle and a multitude of other processes that are germane to tumor development. The functional inactivation of RB has been recognized to occur sporadically in a large fraction of human tumors where it is believed to contribute to disease initiation and/or progression. Several studies have now shown that the specific loss of this tumor suppressor represents a selective vulnerability that could be targeted therapeutically and therefore provide a precision approach to exploiting RB loss [1-5].

**Figure 1:**
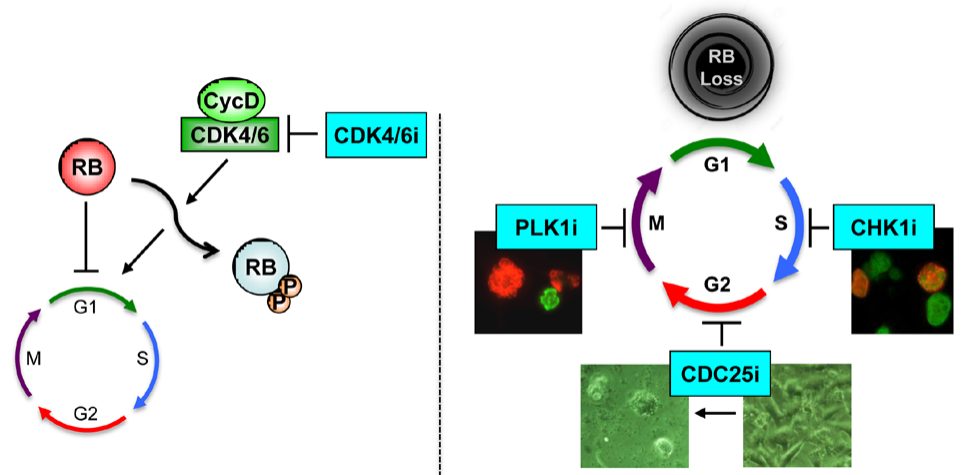
Left, RB-positive tumor cells respond to CDK4/6 inhibitors (CDK4/6i) **Right**, RB deficiency, which frequently occurs in TNBC accelerates cell proliferation and renders cells resistant to CDK4/6i. However, these tumors are highly sensitive to inhibitors of: CDC25, which induce cell death; CHK1, which lead to increased replication driven strand breaks; and PLK1, which promote mitotic catastrophe (Refs. 2 and 5).

**RB-pathway and breast cancer**: Breast cancer is a heterogeneous disease, wherein different manifestations impact prognosis and standard of care regimens. Typically, the presence of estrogen receptor (ER) and progesterone receptor (PR) or epidermal growth factor receptor 2 (HER2) amplification status delineates a course of treatment. Tumors that are HER2 positive are treated with drugs that target this oncogene (e.g. trastuzumab or lapatininb), while ER/PR positive tumors can be treated based on dependence on estrogen (e.g. tamoxifen or letrozole). Tumors that lack ER/PR and HER2 are termed triple-negative breast cancer (TNBC) and are treated systemically with chemotherapy due to the absence of a defined target for therapeutic intervention.

RB is believed to be inactivated as a result of two different mechanisms in breast cancer [6]. 1. RB gene loss, typically as a result of homozygous deletion, occurs predominantly in triple negative breast cancer. This event is relatively rare in ER/PR or Her2 positive cancers at diagnosis. However, in the metastatic setting following treatment with endocrine therapy there is selection for increased loss of RB [7]. 2. RB can also be inactivated by phosphorylation that is initiated by CDK4/6 containing complexes. In breast cancers, amplification of the positive regulators Cyclin D1 and CDK4/6, or loss of the negative regulator p16^ink4a^ are all known to occur [8]. Additionally, a plethora of other events can lead to aberrant CDK4/6 activity that deregulates the normal controls over RB phosphorylation. The importance of CDK4/6 as a therapeutic target is now well-established in ER/PR positive breast cancer where palbociclib, ribociclib and abemaciclibe are all FDA approved. Even though these agents are effective, it is clear that disease progression can occur and this is associated with the selection for RB loss [9].

Thus, defining means to selectively target RB loss could represent a new targeted approach for TNBC, and could represent an important avenue for the treatment of ER/PR positive tumors that progress on CDK4/6 inhibitor treatment.

**Selective targeting of RB loss:** Two recent studies have provided new insights into how the loss of RB could be exploited as a unique vulnerability in breast cancer [2, 5]. Both studies used a combination of drug screens and functional studies coupled with the analysis of clinical populations to credential drug targets and delineate mechanisms of therapeutic sensitivity.

The study from Witkiewicz et al. began with the premise of defining drugs that were specifically modified by the activation status of RB. RB can be activated with CDK4/6 inhibitors and therefore screens were performed in RB-proficient models identifying drugs where cytotoxicity was antagonized by CDK4/6 inhibition. Parallel screens were carried out with panels of TNBC cell lines that had either intrinsically different RB-status, or matched models where RB had been selectively abrogated with CRISPR or ShRNA approaches. From a large number of drugs, essentially three classes of targeted drugs emerged from this investigation: 1. CHK1 inhibitors; 2. PLK1 inhibitors; and 3. Aurora Kinase inhibitors. Importantly, each of these kinases is expressed at higher levels in RB-deficient tumors. Mechanistic analysis suggested that RB loss contributes to sensitivity to CHK1 and PLK1 through different mechanisms. In the case of CHK1, RB loss allows for more DNA replication to occur in the presence of replication stress that translated into more DNA damage and cell death. In the case of PLK1, RB loss allows for ongoing DNA replication in spite of the block in mitosis, leading to increased DNA ploidy and more catastrophic mitotic events. Both of these endpoints are blocked by the activation of RB, which prevents the ongoing DNA replication. In xenograft models, RB deficient tumors were more sensitive to CHK1 inhibition.

Liu et al. performed focused drug screens to identify inhibitors that could target TNBC cells with mutations in RB1, PTEN and/or TP53, as these tumor suppressors are frequently lost together in this aggressive subtype. Screens of primary Rb/p53-deficient and Pten/p53-deficient mammary tumors from mouse models of TNBC, as well as on established RB1/PTEN/TP53 mutant human TNBC lines identified the dual CDC25 phosphatase as a common target. Expression and activity of CDC25 are stimulated in TNBC at the transcription level through loss of RB1, PTEN and TP53 as well as through multiple post-translational modifications. Both pharmacological and genetic inhibitors of CDC25 induced cell death in diverse RB1-negative and RB1-positive TNBC lines, the latter of which included lines that were refractory to CDK4/6 inhibitors. CHK1 induces checkpoint arrest by blocking CDC25 and activating WEE1 kinase, thereby subduing CDK1 activity and the G2 to M phase transition. Importantly, CDC25 and WEE1 inhibitors synergized to kill TNBC, indicating that these factors promote cell demise via distinct mechanisms. Indeed, CDC25 and WEE1 inhibitors had different effects on cell cycle progression in TNBC cells. Prolonged CDC25 suppression led to induction of PI3K signaling (pSer473AKT/PKB), a likely feedback mechanism that sustains survival. Accordingly, CDC25 antagonists cooperated with PI3K inhibitors to effectively attenuate growth of TNBC in xenograft models.

**Clinical implications:** The preclinical data that has emerged suggests that existing drugs that disrupt the CHK1/CDC25/WEE1/CDK1 and G2/M checkpoint control could be deployed in a targeted fashion against tumors that have lost RB. Well-designed clinical trials are now required to prove that these specific vulnerabilities of RB loss are clinically actionable. An interesting approach would be to employ an unselected TNBC patient population, where it would be expected that a significant number of patients (25-30%) would have RB deficient disease. This would provide a highly controlled setting where the impact of RB-status on therapeutic response could be directly interrogated and serve as the basis for future prospective patient selection. In spite of the promise of genetically targeted interventions, given the heterogeneity of most solid tumors, optimism should be tempered. It will be critical to consider multi-agent combinations that are synergistically active against RB-deficient tumors, perhaps exploiting multiple features of the RB loss state to most effectively intercede.
